# Bis-benzylidine Piperidone RA190 treatment of hepatocellular carcinoma via binding RPN13 and inhibiting NF-κB signaling

**DOI:** 10.1186/s12885-020-06896-0

**Published:** 2020-05-06

**Authors:** Ruey-Shyang Soong, Ravi K. Anchoori, Richard B. S. Roden, Rou-Ling Cho, Yi-Chan Chen, Sheng-Chieh Tseng, Yun-Li Huang, Po-Cheng Liao, Yu-Chiau Shyu

**Affiliations:** 1 Department of General Surgery, Keelung Chang Gung Memorial Hospital, Keelung, Taiwan; 2 Chang Gung Medical College Taoyuan, Taoyuan, Taiwan; 3 Community Medicine Research Center, Keelung Chang Gung Memorial Hospital, No.200, Ln 208, Jijin 1st Rd, Anle Dist, 204, Keelung City, Taiwan R.O.C; 4 Department of Oncology, Johns Hopkins University, Baltimore, MD USA; 5 Department of Pathology, Johns Hopkins University, Baltimore, MD USA; 6 Department of Nursing, Chang Gung University of Science and Technology, Taoyuan, Taiwan; 7 Institute of Molecular Biology, Academia Sinica, Taipei, Taiwan

**Keywords:** Apoptosis, Hepatocellular carcinoma, NF-κB, Proteasome inhibitor, RA190

## Abstract

**Background:**

According to GLOBOSCAN, hepatocellular carcinoma (HCC) claimed 782,000 lives in 2018. The tyrosine kinase inhibitor sofafenib is used to treat HCC, but new anticancer agents targeting different pathways are urgently needed to improve outcomes for patients with advanced disease. The aberrant metabolism and aggressive growth of cancer cells can render them particularly susceptible to proteasome inhibition, as demonstrated by bortezomib treatment of multiple myeloma. However, resistance does emerge, and this 20S proteasome inhibitor has not proven active against HCC. The bis-benzylidine piperidone RA190 represents a novel class of proteasome inhibitor that covalently binds to cysteine 88 of RPN13, an ubiquitin receptor subunit of the proteasome’s 19S regulatory particle. RA190 treatment inhibits proteasome function, causing rapid accumulation of polyubiquitinated proteins. Considerable evidence suggests that nuclear factor κB (NF-κB) signaling, which is dependent upon the proteasome, is a major driver of inflammation-associated cancers, including HCC.

**Methods:**

Human HCC cell lines were treated with titrations of RA190. The time course of endoplasmic reticulum stress and NF-κB-related mechanisms by which RA190 may trigger apoptosis were assessed. The therapeutic activity of RA190 was also determined in an orthotopic HCC xenograft mouse model.

**Results:**

RA190 is toxic to HCC cells and synergizes with sofafenib. RA190 triggers rapid accumulation of polyubiquitinated proteins, unresolved endoplasmic reticulum stress, and cell death via apoptosis. RA190 blocks proteasomal degradation of IκBα and consequent release of NF-κB into the nuclei of HCC cells. Treatment of mice bearing an orthotopic HCC model with RA190 significantly reduced tumor growth.

**Conclusions:**

RA190 has therapeutic activity in a xenograft model, and with sorafenib exhibited synergetic killing of HCC cells in vitro, suggesting further exploration of such a combination treatment of HCC is warranted.

## Background

Hepatocellular carcinoma (HCC) is the fifth most common malignancy and the third leading cause of cancer mortality worldwide [1]. The Barcelona Clinic Liver Cancer Staging (BCLC) treatment guidelines recommend surgical resection, liver transplantation, or radio frequency ablation for the management of early stage of HCC, whereas transarterial chemoembolization or systemic chemotherapy are used for patients with intermediate or advanced stage HCC [2]. Systemic chemotherapy for HCC is limited to only a few conventional drugs (Sorafenib [3, 4], regorafenib [5], lenvatinib, cabozantinib and ramucirumab) with unsatisfactory objective response rates [6, 7]. These drugs target multiple tyrosine kinase pathways with the exception of ramucirumab that targets VEGF-R2. Even immunotherapy (nivolumab) targeting programmed cell death protein-1 (PD-1) exhibits only a 15–20% objective response rate in advanced HCC [8]. Thus, new anticancer agents with a distinct mechanism of action are urgently needed to improve outcomes for advanced HCC patients.

The ubiquitin-proteasome system (UPS) is a complex and tightly controlled system that mediates protein homeostasis by degrading 80–90% of cellular proteins and it is central to regulating key cellular functions through targeted protein degradation. The high metabolic and protein synthesis rates that sustain the aggressive growth of cancer cells place increased burden on the UPS, thereby creating a therapeutic window. Furthermore, several driver oncogenic pathways are dependent on the UPS to mediate signaling, providing further rationale to UPS inhibition as cancer therapy [9]. Indeed, the 20S proteasome subunit has been validated as a therapeutic target in cancer by the licensure of three PSMB5-targeted inhibitors, beginning in 2003 with bortezomib for the treatment of multiple myeloma. Recently, accumulating evidence suggests that small molecules targeting alternative components of the UPS, including the receptors for its ubiquitinated substrates, also have potential for the treatment of HCC [10].

The bis-benzylidine piperidone RA190 covalently binds to cysteine 88 of RPN13 (also called ADRM1), a key ubiquitin receptor in the 19S regulatory particle of the proteasome, and inhibits its function causing rapid accumulation of polyubiquitinated proteins. RA190 showed a therapeutic effect in multiple myeloma, cholangiocarcinoma [11], ovarian and cervical cancer models in previous studies [12]. In this study, we examine the impact of RA190 on HCC cells, including the levels of polyubiquitinated proteins, Endoplasmic Reticulum (ER) stress, apoptosis, and its therapeutic potential against an orthotopic xenograft model. Since the tyrosine kinase inhibitor sorafenib is used to treat HCC, we also examine whether combining RA190 with sorafenib has a synergistic antitumor effect, given their distinct mechanisms of action.

Nuclear factor κB (NF-κB) is important for promoting inflammation-associated cancer [13, 14]. Its activation triggers the transcription of numerous genes with κB binding sites, most of them are involved in the regulation of inflammation, immune responses and cell survival [15]. IκBα is the most prominent IκB family member bound to NF-κB, restricting it to the cytoplasm and thereby inhibiting its activity. Phosphorylation of IκBα by the IKK complex at two different serine residues (Ser32 and Ser36) marks IκBα for subsequent degradation through an ubiquitin-dependent pathway in the proteasome, thereby liberating NF-κB for nuclear entry, DNA-binding and transcriptional activation [16]. Inhibition of IκBα degradation by proteasome inhibitors prevents the release and nuclear entry of NF-κB. RA190 was previously reported to block NF-κB signaling via stabilization of p-IκBα [11, 12]. Here, we examine whether RA190 inhibits the NF-κB pathway by preventing IκBα degradation by the proteasome, causing the cytoplasmic accumulation of NF-κB in HCC cells.

## Methods

### Cell lines

HepG2, human hepatocellular carcinoma cell lines were purchased from Bioresource Collection and Resource Center (BCRC numbers RM60025 and 60,434), Taiwan and were grown in DMEM medium (Gibco) supplemented with 10% FBS (Gibco), 100 μg/mL penicillin (Gibco), and 100 U/mL streptomycin (Gibco).

### Animal

Mouse experiments were conducted with the prior ethics approval from Animal Care and Use Committee in Chang Gung Memorial Hospital, Keelung. Male nude mice CAnN.Cg-Foxn1nu/CrlNarl (4–6 weeks old, ~ 20 g) were purchased from the National Laboratory Animal Center (Taipei, Taiwan). All mice were sacrificed by using CO_2_ for 20 min. All animal procedures were conducted in Abnova Company and were performed according to approved protocols and by recommendations for the proper use and care of laboratory animals.

### Chemicals

RA190 and RA190B were synthesized in-house and purified to 99% as previously described [12]. Sorafenib and bortezomib were purchased from L.C. laboratories.

### qRT-PCR

Total RNA was isolated from cells using the RNeasy mini kit (Qiagen). Extracted RNA was normalized for concentration and reverse transcribed using iScript cDNA synthesis kit (Bio-Rad). Taqman gene expression assays measured *Bip-1, ATF-4, CHOP10, RPN13, iNOS, GAPDH* expression levels utilizing Taqman gene expression master mix (Applied Biosystems) per the manufacturer’s instructions. Spliced *XBP1* mRNA was assayed with SsoFast™ EvaGreen® Supermix (Bio-rad) following the recommendations for the iCycler System. Forward and reverse primer was: 5′-TGCTGAGTCCGCAGCAGGTG and 5′-TGGGTCCAAGTTGTCCAG AATGCC. Calculations were done according to the Livak method and normalized to reference gene *GAPDH*. Each condition was replicated three times; each sample was run in triplicate. 2 μL cDNA sample was used for PCR amplification with iQ™ SYBR® Green Supermix (Bio-Rad) according to the manufacturer’s protocol.

### Biotin labeling assay

HepG2 cells (5 × 10^6^) were lysed using MPER (Pierce) lysis buffer (1 mL) according to the manufacturer protocol. Cell lysates were centrifuged at 10,000 rpm briefly (2 min) at 4 °C to remove cell debris. Lysate supernatant (100 μL) was pre-cleared with streptavidin dyna beads (20 μL) for 1 hour at 4 °C to remove non-specific biotin binding and incubated with compounds (indicated concentrations) at 4 °C for 1 h. An equal amount of each sample (20 μL of lysate) was mixed with the same volume of Laemmli sample buffer (20 μL) (BioRad) and was boiled for 5 min. The proteins were separated using 4–15% Bio-Rad Mini-PROTEAN SDS-PAGE gel (1 h at 100 V) and transferred to the membrane overnight at 4 °C (24 V). The membrane was blocked with 5% BSA in PBST (phosphate buffered saline containing 0.1% Tween 20) for 1 h at room temperature and washed for 20 min (3X in PBST). Then the membrane was probed with HRP-streptavidin (1:10,000 in PBST) for 1 h at room temperature and soaked for 30 min (3X in PBST) and developed using HyGLO chemiluminescent detection reagent (Denville) for biotin recognition.

### Western blot analysis

50 μg/well of protein from the HepG2 cell lysate was separate using SDS-PAGE and transferred to a nitrocellulose membrane (G.E. Bioscience). After blocking with 5% skim milk in PBST for 1 h at room temperature, membranes were incubated overnight with primary antibody at 4 °C. Layers were then washed with PBST and incubated with horseradish peroxidase (HRP)–conjugated secondary antibody before visualization with ECL plus (G.E. Bioscience). All antibodies, including NF-κB (Cell signaling D14E12, #Cat.8242), IκBα (Cell signaling L35A5, #Cat. 4814), poly-Ub (Enzo FK2, #Cat. BML-PW0150–0100), P21 (Cell Signaling 12D1, #Cat 2947), Actin (Abcam EPR16769, #Cat. Ab179467), lamin A (Abcam EPR4068, #Cat. ab108922), and tubulin (Abcam EPR13796, #Cat. ab210797) were diluted in 5% BSA buffer. The dilution ratio of each antibody followed the manufacturer’s recommendation.

### Clonogenicity assay

Clonogenicity assays were performed as previously described [17]. Briefly, HepG2 cells (500 cells/well) were plated in a 6-well plate at day 1. Cells were treated on day 2 with RA190 at the indicated concentrations. After 14-days incubation, colonies were stained with crystal violet (0.5% w/v) and imaged. The intensity of the signals were quantified by Image J software V1.50f (National Institutes of Health).

### Immunofluorescence stain

1.5 × 10^4^ cells of HEpG2 per well were seeded in the 8-chamber slide in 500 μL of culture medium, incubated at 37 °C under humidified 95% air/5% CO_2_ for 18 h. After treatment for 30 min, the slides were fixed with 4% paraformaldehyde (in PBS pH 7.4) for 10 min, and permeabilized with 400 μL of 0.1% Triton X100 in 1X PBS at room temperature for 5 min. After blocking with 3 mL 10% donkey serum in PBS pH 7.4 at R.T. 30 min, the slides were incubated overnight with the primary antibody, IκBα (Cell Signaling, #Cat. 4812S), NF-κB (B.D., #Cat 558,393) and proteasome (PSMD2 19S RP subunit (NOVUS, #Cat. NB100–1483)) at 4 °C. After washing slides with PBST pH 7.4, secondary Ab was added (anti-Rabbit-Alexa488 (Invitrogen, #Cat. 412,438), anti-Goat-DyLight650 (Bethyl, #Cat. A50-200D5) and proteasome-anti-Mouse-Alexa546 (Invitrogen, #Cat. 412,438)) in 1% donkey serum in PBS pH 7.4 for 2 h. Finally, the nucleus was stained with DAPI (Thermo, #Cat. D1306) in 1% donkey serum in PBS pH 7.4 for 10 min, and the slides mounted using antifade. The immunofluorescence signal was visualized by confocal microscopy using a Leica TCS SP8 and edited by Leica LAS X software.

### Flow cytometry analysis

Cell were stained with Annexin V-PE (B.D., #Cat. 560,930), propidium iodide (B.D., #Cat. 556,463), or Active Caspase 3-PE (B.D., #Cat. 561,011) and flow cytometric analyses performed on a Becton-Dickinson FACScan with CELLQuest software (Becton-Dickinson Immunocytometry System, Mountain View, CA) and Flowjo 10 software.

### Orthotopic tumor implantation model

Male nude mice, 4–6 weeks old, were anesthetized via *i.p.* administration of 80 mg/kg ketamine and 10 mg/kg xylazine. While under demonstrable anesthesia, an upper midline incision of the abdomen was made and 5 × 10^5^ HepG2-Luc cells mixed with Matrigel (1:1) in 20 μL was injected into the left lobe of the liver through a 23-gauge syringe by laparotomy, six in each group. To avoid the leakage of the tumor and seeding into the peritoneum, the injection site was compressed by cotton swab for 2 min until no bleeding was evident from the liver surface [18]. After tumor injection, the abdominal wound was closed by interrupted stitches afterward afforded analgesia. To monitor the tumor growth in mice, the tumor was imaged by the IVIS system as previously described [19].

### Statistical analysis

All data are expressed as mean ± S.E. where indicated and are representative of at least two separate experiments. In the tumor treatment experiments, the outcome of interest was the volume of the tumor estimated using calipers until euthanasia based on the animal protocol (in stress, weight change greater than 20%). The tumor volumes were compared using ANOVA at each time point. All *p*-values < 0.05 were considered significant. The statistics were calculated by Prism 6 software.

## Results

### RA190 exhibits more potent HepG2 cell killing than sorafenib

**Fig. 1:**
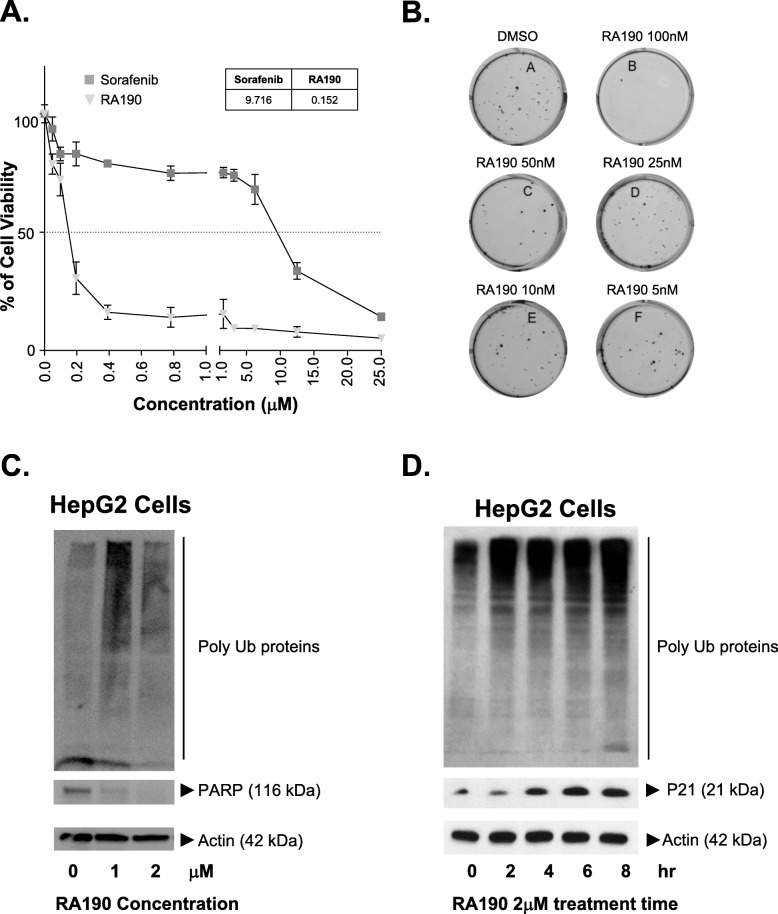
Impact of RA190 upon HepG2 cell viability, colony formation, polyubiquitinated protein, and apoptotic markers. **a** HepG2 cells were plated at 96/well of a microtiter plate in the presence of titrations of RA190 or sorafinib, and after 72 h the cell viability was determined by MTT assay. **b** Treatment of HepG2 cells with RA190 reduced the number tumor colonies in a clonogenicity assay. **c** HepG2 cells after 12 h treatment with 0, 1, or 2 μM RA190 were harvested and subjected to Western blot analysis with antibody to Ubiquitin, PARP and Actin (full-length blots/gels are presented in Fig. S4). **d** HepG2 cells were treated with 2 μM RA190 and harvested at the indicated times. Cell lysates were subjected to Western blot analysis with antibody to Ubiquitin, p21 and Actin (full-length blots/gels are presented in Fig. S5)

HepG2 cells were seeded at a density of 2500 cells/well in 100 μL DMEM medium supplemented with 10% FBS in 96-well plate. Twenty-four hours post seeding, the cells were treated with RA190 and sorafenib at specified concentrations. Seventy-two hours after treatment, cells were incubated according to the manufacturer’s protocol with the MTT reagent for 1 h, and absorbance at 570 nm measured to assess inhibition of cell growth. The IC_50_ for RA190 of 0.15 μM was significantly lower than for sorafenib (9.7 μM) against HepG2 cells (Fig. 1a). In a clonogenicity assay, HepG2 cells treated with RA190 exhibited a reduced number of tumor colonies with an IC_50_ of 0.1 μM (Fig. 1b).

### RA190 triggers rapid accumulation of polyubiquitinated proteins

Since RA190 is a 19S RP-targeted proteasome inhibitor [12], we examined its impact on the levels of polyubiquitinated proteins in HepG2 cells by ubiquitin immunoblot analysis. RA190 treatment of HepG2 cells for 12 h at 1 μM or 2 μM dramatically increased the levels of polyubiquitinated proteins and in a dose-dependent manner (Fig. 1c and d). We also observed a significant increase in insoluble polyubiquinated cellular proteins in the lysates removed by high speed centrifuging before loading into the gel. This likely accounts for the reduction in ubiquitinated protein observed by Western blot when treating with higher doses of RA190 (lane 3 versus lane 2). Because RPN13 also acts to promote UCH37’s deubiquitinase function, the molecular weight of the accumulated polyubiquitinated proteins observed following exposure to RA190 was higher than seen in bortezomib-treated cells, as previously described [20].

### RA190 binds to RPN13 in HepG2 cells

**Fig. 2:**
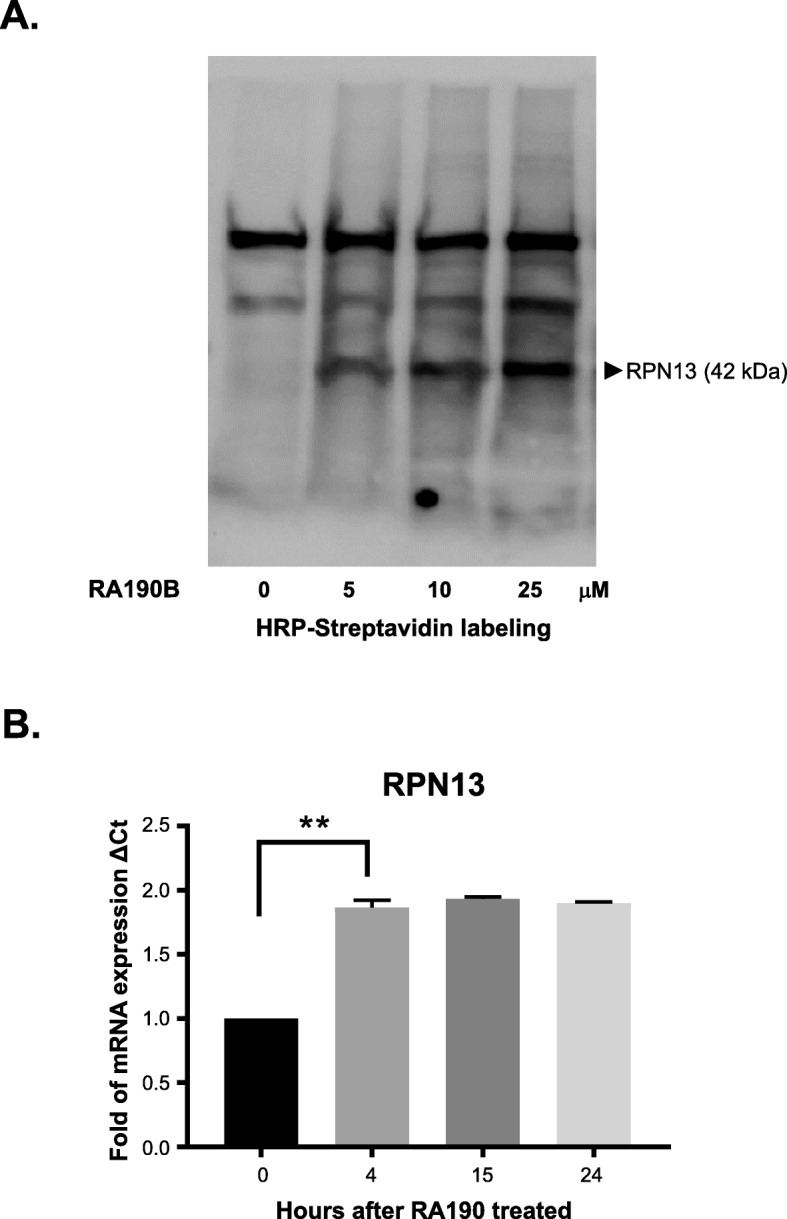
RA190B binds to RPN13 in HepG2 cells. **a** HepG2 cell lysates were incubated with 0, 5, 10 or 25 μM RA190B, separated by SDA-PAGE, transferred to a membrane and probed with HRP-streptavidin. After development using chemiluminescence, a prominent band at 42 KDa was evident, a molecular weight consistent with RPN13 (full-length blots/gels are presented in Fig. S6). **b** RPN13 (*ADRM1*) mRNA expression as determined by quantitative RT-PCR was significantly increased after 4 h RA190 treatment

To identify RA190’s cellular target in HepG2 cells, biotin was covalently linked to RA190 via its free amine functionality (RA190B), as previously described [12]. HepG2 cell lysate was treated with RA190B (at 0, 5, 10, or 25 μM), subjected to SDS-PAGE, and probed with streptavidin-peroxidase following protein transfer to a polyvinylidene difluoride (PVDF) membrane. The streptavidin-peroxidase bound to biotinylated cellular proteins and a new band at 42 kDa was found in treated samples (Fig. 2a) that is consistent with the molecular weight of RPN13 and our previous data in other cancer cell lines [12]. In addition, mRNA collected from HepG2 cells was treated with RA190 at either 0 or 2 μM for 0, 4, 15, or 24 h, was subjected to quantitative RT-PCR with primers specific for *ADRM1*, the gene encoding RPN13, and the housekeeping gene *GAPDH*. The *ADRM1* mRNA expression was significantly increased by RA190 treatment (Fig. 2b) relative to the housekeeping gene. Taken together, the results suggest that RA190 binds to and the 42 kDa RPN13 protein in HepG2 cells, triggering compensatory upregulation of *ADRM1*.

### Rapid accumulation of polyubiquitinated proteins leads to ER stress and apoptosis

**Fig. 3:**
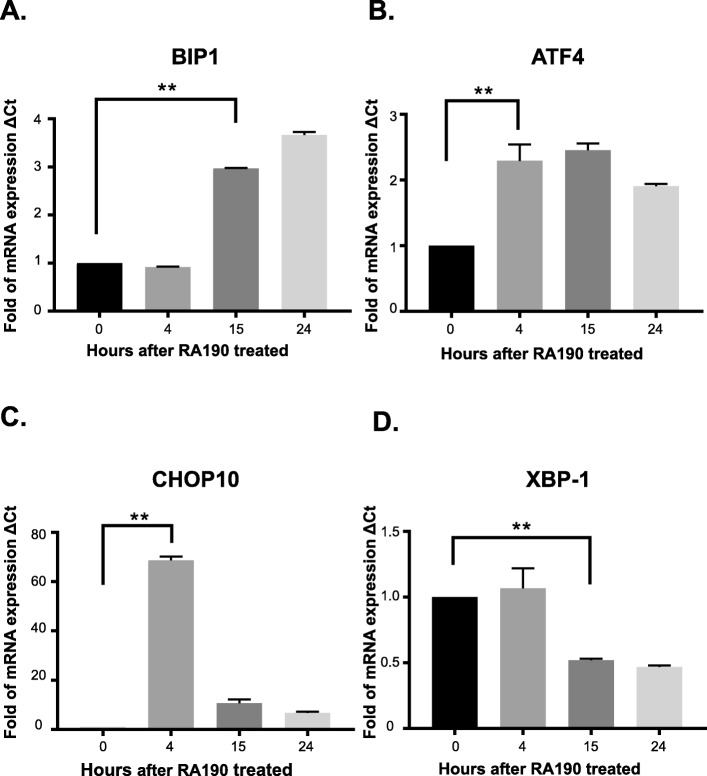
Elevation of mRNA levels of UPR genes after RA190 treatment. **a, b, c, d** The mRNA expression levels of ER stress proteins BIP-1, ATF-4, CHOP10, and spliced XBP-1 were determined by quantitative RT-PCR in HepG2 cells 0, 4, 15 and 24 h after 2 μM RA190 treatment

**Fig. 4:**
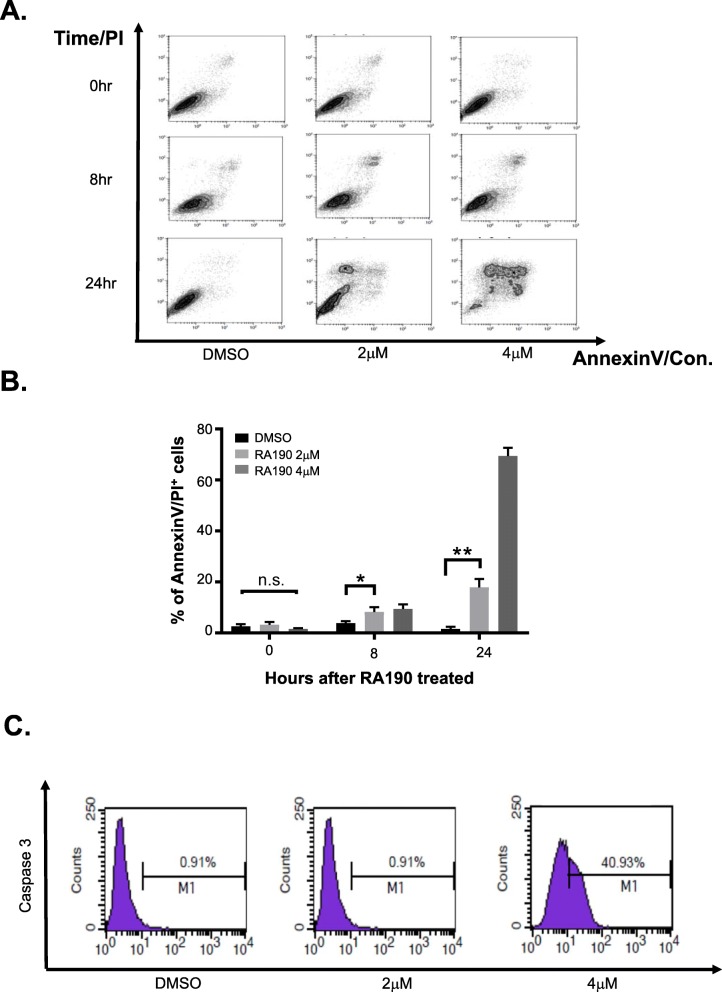
RA190 triggers apoptosis in HepG2 cells. **a, b** The percentage of PI^+^/Annexin V^+^ HepG2 cells was determined by flow cytometry after treatment with 0, 2 or 4 μM RA190 for 0, 8 or 24 h. **c** Active Caspase 3 level was determined by flow cytometry after treatment with 0, 2 or 4 μM RA190 for 8 h

In addition to the rapid accumulation of polyubiquitinated unfolded proteins, RA190 treatment also triggered the elevation of *BIP-1*, *ATF-4*, *CHOP10* and spliced *XBP-1* transcript expression levels (Figs. 3a-d), consistent with an ER stress response. At later time points after RA190 treatment, HepG2 cells also exhibited a significantly increased the proportion of Annexin V/PI double positive cells (Figs. 4a-c), suggesting activation of apoptosis because of an unresolved ubiquitin proteasome stress response. Indeed, caspase 3 (Fig. 4c) and PARP (Fig. 1c) cleavage and p21 expression (Fig. 1d) were also considerably increased in HepG2 cells after RA190 treatment, providing further biochemical evidence of the activation of apoptosis.

Autophagy is a potentially compensatory pathway to mitigate the impact of proteasome inhibition. Formation of the lipidated LC3-II, a biomarker of autophagy, was not elevated within 8 h after RA190 2 μM treatment (Fig. S1), although this was seen upon addition of 10 μM chloroquine, a positive control. Taking together, the rapid accumulation of polyubiquitinated proteins after RA190 treatment caused ER stress that could not be counteracted by the induction of autophagy, leading to apoptosis of the HepG2 cells.

### RA190 blocks IκBα degradation and limits NF-κB entry to the nucleus

**Fig. 5:**
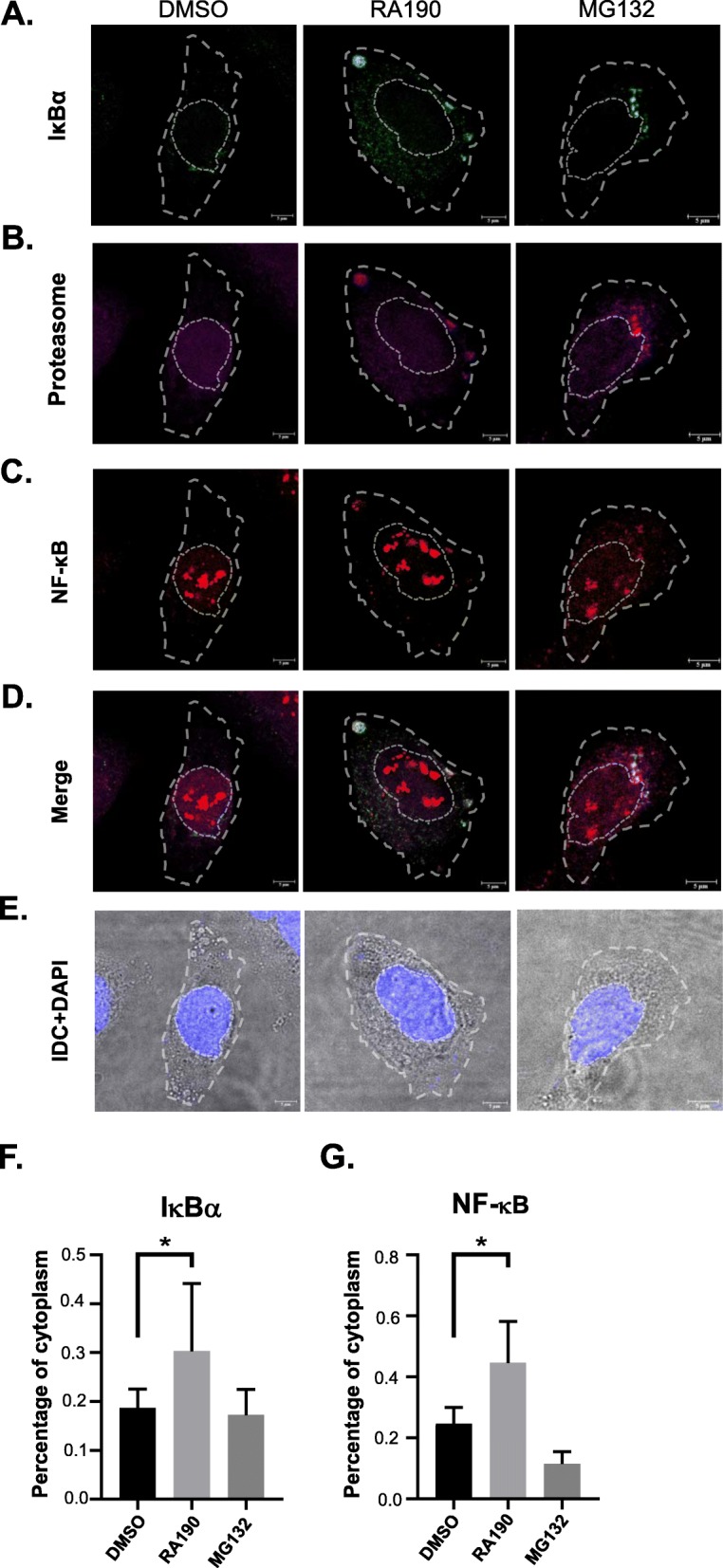
RA190 causes accumulation of IκBα and NF-κB in the cytoplasm, co-localized with proteasomes. HepG2 cells were treated with DMSO, 2 μM RA190 for or 25 μM MG132 for 30 min. The cells were fixed, permeabilized and stained for IκBα **a**, proteasome **b**, NF-κB **c** and viewed by confocal fluorescence microscopy individually, or **d** merged, or **e** under phase contrast. **a, b, c** IκBα and NF-κB in RA190 and MG132-treated cells is significantly accumulated in cytoplasm, and apparently co-localized with the proteasome. The proportion of fluorescence in the cytoplasm versus the nucleus was quantified for **f** IκBα, or **g** NF-κB

**Fig. 6:**
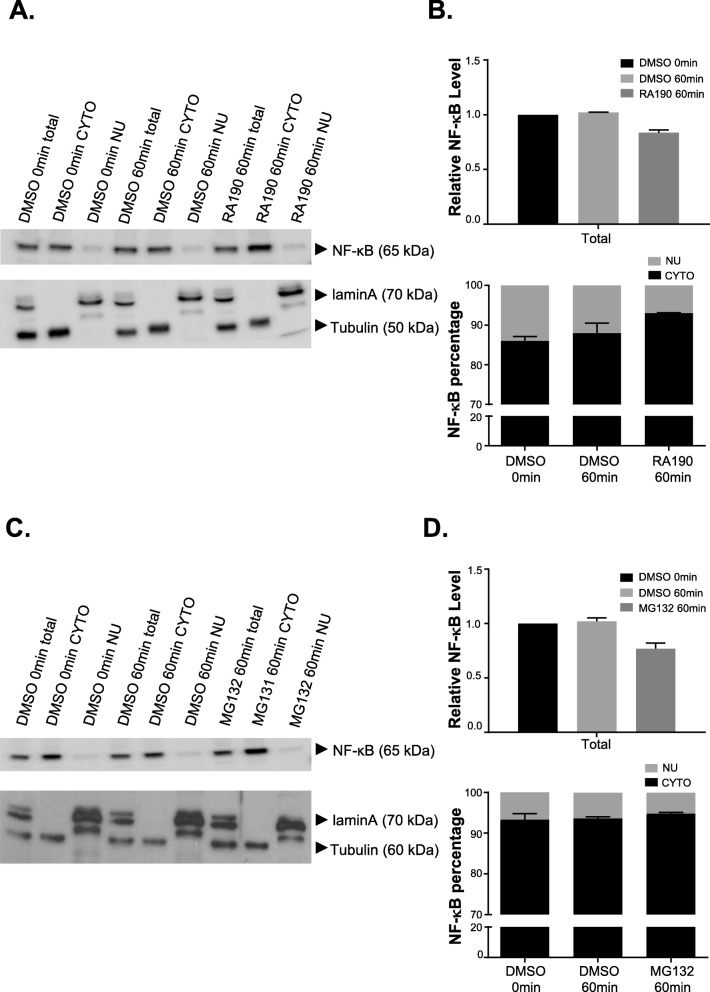
NF-κB is significantly accumulated in the cytoplasmic fraction after RA190 treatment. HepG2 cells were treated with DMSO or 2 μM RA190 for 60 min, harvested and lysed (total fraction). Cytoplasmic and nuclear fractions were separated, and each fraction was subjected to Western blot with antibody to NF-κB, lamin **a** (a nuclear marker) and tubulin (a cytoplasmic marker). **a** NF-κB showed significant accumulation in the cytoplasmic fraction of cells when treated with RA190 for 60 min, as compared with DMSO (full-length blots/gels are presented in Fig. S7). The quantified data is presented in a bar graph **b**. **c** Similar results were obtained with MG132 treatment (full-length blots/gels are presented in Fig. S8) and the quantification is presented in a bar graph **d**

To examine whether RA190 blocked IκBα degradation and thereby the entry of NF-κB to the nucleus, we used immunofluorescence to visualize IκBα and NF-κB at 30 min after treating with RA190 or the 20S proteasome inhibitor MG132 (which has a similar action as bortezomib) as compared to DMSO (vehicle)-treated cells. IκBα was readily detectable in the cytoplasm of RA190 or MG132-treated cells (Fig. 5 and Fig. S2). In the DMSO treated cells, IκBα was almost undetectable, consistent with its rapid degradation by the proteasome. Most of the IκBα is co-located with the proteasome (Fig. 5b) in RA190 or MG132-treated cells. In the DMSO-treated group, the majority NF-κB protein was nuclear. While much was still in the nucleus, the NF-κB protein was significantly increased in the cytoplasm in the RA190 treated group (Fig. 5c), likely reflecting the short incubation period. The IκBα signal intensity in the cytoplasm and nuclei was quantified by Image J software and this analysis showed the fraction in the cytoplasm was significantly higher in RA190-treated group (Fig. 5f). Likewise, the percentage of NF-κB signal intensity in cytoplasm was also higher in RA190-treated group (Fig. 5g). This result was also examined 60 min post-treatment by immunoblot of the cytoplasmic vs. nuclear cellular factions, and a similar pattern was observed (Fig. 6). The NF-κB was significantly accumulated in the cytoplasm at 60 min after RA190 treatment (Fig. 6a-b), and a similar finding was evident in MG132-treated HepG2 cells (Fig. 6c-d). The levels of phosphorylated and ubiquitinated IκBα also built up after RA190 and bortezomib treatment (Fig. S3). When considered together, these observations suggest RA190 prevents IκBα degradation by the proteasome.

### RA190 treatment inhibits growth of an orthotopic HCC xenograft model

**Fig. 7:**
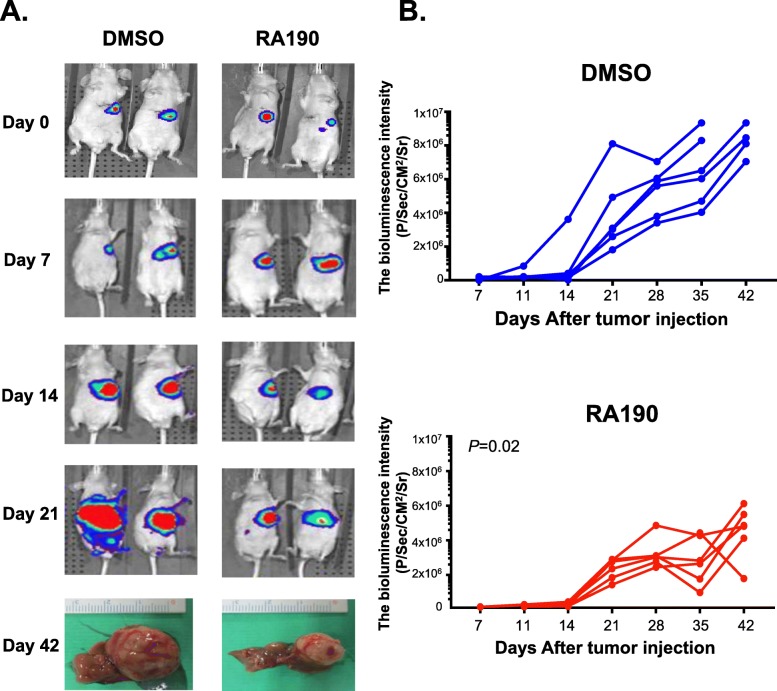
RA190 treatment inhibited the growth of orthotopic HCC tumor model. **a** Bioluminescence imaging showing the HepG2 tumors following orthotopic challenge with HepG2-Luc cells (5 × 10^5^ /mouse) administered in the left lobe of liver of nude mice. Mice were treated with either DMSO or RA190 (20 mg/kg) once daily via the intra peritoneal route beginning at day 7 and ending at day 28. Imaging of bioluminescence after injection of luciferin via the intraperitoneal route was performed on an IVIS 200 imager and the data plotted **b**. Bioluminescence was significantly different (*P* < 0.02) between the groups on day 42

HepG2-Luc cells (5 × 10^5^ cells in 20 μL) were injected into the left lobe of the liver at day 0. Once the tumor signal was detected at day 7 upon *i.p.* injection of luciferin and IVIS imaging, the mice were randomized into two groups (6 mice in each group). Upon randomization, treatment (RA190 20 mg/kg in the active arm, and DMSO in the control arm, intraperitoneal injection) was initiated once daily for 21 days. The tumor was visualized and bioluminescence quantified after injection of luciferin by the IVIS imaging system again at day 11, 14, 21, 28, 35 and 42. Two mice in the DMSO group were sacrificed early due to tumor burden at day 35. Surviving mice in both groups were sacrificed at day 42 and the tumor volume was smaller in the liver specimen of the RA190-treated mice (Fig. 7a). Figure 7b shows the signal change in individual mice. The bioluminescence intensity in RA190 groups was significantly lower than the DMSO group (*P* = 0.02) at day 35 time point.

### RA190 and Sorafenib combination is synergistic

**Fig. 8:**
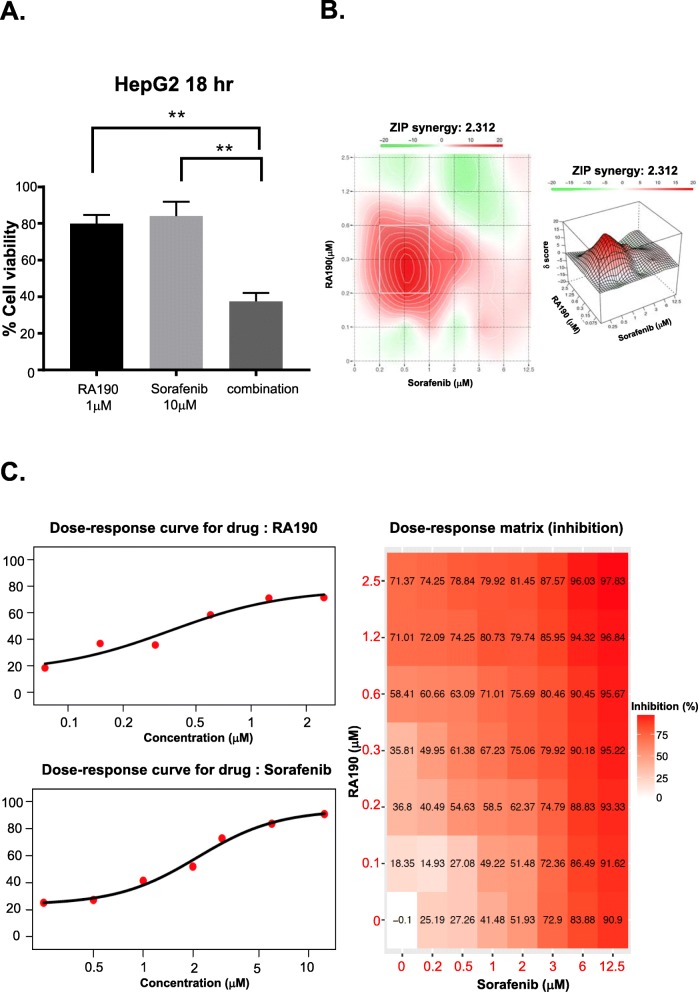
RA190 combination with Sorafenib showed synergy against HepG2 cells. **a** Bar graph depicting HepG2 cell viability after 48 h of each treatment regimen. To seek the conditions of optimal synergy, a checkerboard analysis was performed using titrations of RA190 and Sorafenib. **b, c** A surface plot of the Synergy score showed the most synergistic cell killing occurred at 0.3 μM RA190 and 0.5 mM Sorafenib

Synergistic combinations of multiple drug treatments targeting different pathways have proven most effective to treat cancer and stave off the emergence of resistance. Zero interaction potency (ZIP) score is an approach used to assess synergy and it captures the drug interaction relationships by comparing the change in the potency of the dose–response curves between individual drugs and their combinations [21]. To test whether combination treatment might have a synergetic killing effect for HCC, we reduced the RA190 concentration to 1 μM and Sorafenib to 10 μM. After treating 18 h, cell viability was still around 80% with individual drugs. However, combining RA190 and Sorafenib, significantly improved the killing effect and cell viability dropped lower than 40% (Fig. 8a). The optimal combination ratios were further sought using a checkerboard analysis to assess cell viability with titrations of RA190 and sofeninib, and analyzing the data using the ZIP synergy score prediction model with the Synergy Finder application. In this experiment HepG2 cells were first treated with Sorafenib and 48 h later with RA190 (for the final 24 h) for a total assay time of 72 h. A ZIP synergy score of 2.31 was achieved, which indicates a synergetic effect (Fig. 8b, c) [21].

## Discussion

The ubiquitin-proteasome pathway is a central component in maintaining protein homeostasis. The increased metabolism of cancer cells is associated with aberrant protein expression, including the accumulation of misfolded or deleterious proteins. This proteotoxic stress renders cancer cells more reliant upon proteasome function to degrade the over-produced/mis-folded protein accumulation, thereby creating a therapeutic window and providing a rationale for proteasome inhibition as a means to shift this delicate equilibrium towards cell death [22]. Proteasome inhibitors have also been examined as potential HCC therapy, and while some preclinical studies showed promising results [23], unfortunately, clinical responses have been less encouraging [24, 25]. This may reflect limited uptake of the licensed proteasome inhibitors by solid HCC tumors, suggesting that testing alternative small molecule proteasome inhibitors is warranted.

NF-κB acts as a central link between hepatic injury, fibrosis, and HCC, and it likely represents an important target for the treatment of liver fibrosis and/or HCC [13]. The activation of NF-κB drives transcription of hundreds of genes with κB binding sites that are involved in the regulation of inflammation, immune responses and cell survival [15]. NF-κB is held in an inactive cytoplasmic complex with an inhibitory IκB protein. To activate NF-κB, the phosphorylated IκBα is detached from the NF-κB complex and transferred to the proteasome for degradation. The malfunction of IκBα is an important driver of aberrant constitutive NF-κB in solid tumors [26], including HCC [14]. NF-κB activity is dependent on the RPN13-UCH37 axis in the proteasome because it contributes to the recognition of polyUb-tagged IκBα as a substrate for the proteasome. Removal of the polyUb tag (deubiquitination) occurs ahead of substrate transfer to the catalytic subunit for degradation. Upon substrate recognition, RPN13 activates the deubiquitinase function of its binding partner UCH37. Therefore, inhibiting RPN13 function blocks both substrate recognition and deubiquitination activity, and this has been shown critical for NF-κB activation via RPN13 knockdown and RPN13 inhibitors such as RA183 and RA190 [27].

Overexpression of *ADRM1* mRNA and /or RPN13 was reported in many types of malignancy, including multiple myeloma, diffuse large B-cell lymphoma, gastric cancer, ovarian cancer, and intrahepatic cholangiocarcinoma and high expression of RPN13 correlated with poor prognosis [11]. The overexpression of *ADRM1* mRNA and RPN13 was detected in HCC, and NF-κB activity was reduced therein by knockdown of *ADRM1*/RPN13 [28]. We further observed that RA190 inhibited IκBα protein degradation and led to the accumulation of IκBα coincident with the proteasome (Fig. 5a, b), consistent with a role for RPN13 in control of NF-κB activity. Indeed, RA190 limits nuclear entry of NF-κB (Fig. 4c, Fig. 5) and inhibition of this pathway likely contributes to HCC cell death. Indeed, we found that RA190 treatment exerted a significant therapeutic effect on an orthotopic HCC xenograft model, as seen in other cancer types [12]. Consistent with an on-target effect, biotinylated RA190 also bound with specificity to the 42 kDa RPN13 in HepG2 cells. RA190 treatment also led to the rapid accumulation of poly-ubiquitinated proteins in HCC cells, and initiated apoptosis (Fig. 4) that is consistent with inhibition of proteasome function and unresolved proteotoxic stress. While RPN13 has previously been seen to regulate cisplatin-induced autophagy, RA190 did not activate the autophagy pathway (Fig. S1) in HCC. While RA190 had a significant tumor control effect in the orthotopic tumor implant model in vivo *(*Fig. 6), it was not curative. This suggests the need for combination therapy by targeting another independent cell pathway critical to HCC viability, such as tyrosine kinase signaling. Sorafenib is used clinically to treat HCC, and we observed synergy with RA190 in vitro.

## Conclusions

In summary, RA190 binds to RPN13 in HCC cells and inhibits proteasome function therein. This triggers apoptosis in the HCC cells because of a rapid accumulation of poly-ubiquinated protein accumulation and resultant unresolved endoplasmic reticulum stress, as well as the inhibition of NF-κB, a critical oncogenic signal in HCC, by preventing the degradation of IκBα. While RA190 treatment slowed the growth of an orthotopic HCC xenograft model, synergy seen in studies in vitro suggest that an RPN13 inhibitor like RA190 might fruitfully be combined with sorafenib as a salvage therapy for HCC patients.

## Supplementary information

**Additional file 1: Figure S1.** RA190 treatment HepG2 cell lines did not activate the autophagy pathway. **Figure S2.** Immunostaining of NF-κB and IκB after treating with RA190. **Figure S3.** RA190 treatment of HepG2 cells produced significant accumulation of phosphorylated/ubiquitinated IκBα.

**Additional file 2: Figure S4.** Original images to Fig. 1C. **Figure S5.** Original images to Fig. 1D. **Figure S6.** Original images to Fig. 2A. **Figure S7.** Original images to Fig. 6A. **Figure S8.** Original images to Fig. 6C. **Figure S9.** Original images to Fig. S1. **Figure S10.** Original images to Fig. S3.

## Data Availability

The data generated and analyzed during the current study are not publicly available due to confidentiality requirements but are available from the corresponding author on reasonable request.

## Abbreviations

HCC: Hepatocellular carcinoma
NF-κB: Nuclear factor-κB
BCLC: Barcelona Clinic Liver Cancer Staging
PD-1: Programmed cell death protein-1
UPS: Ubiquitin-proteasome system
