# Supplementary material for: Bis-benzylidine Piperidone RA190 treatment of hepatocellular carcinoma via binding RPN13 and inhibiting NF-κB signaling
Source: BMC Cancer. 2020 May 6;20:386. doi: 10.1186/s12885-020-06896-0 (PMC7201939; doi:10.1186/s12885-020-06896-0)
Supplement: Supplementary file 1 — Additional file 1: Figure S1. RA190 treatment HepG2 cell lines did not activate the autophagy pathway. Figure S2. Immunostaining of NF-κB and IκB after treating with RA190. Figure S3. RA190 treatment of HepG2 cells produced significant accumulation of phosphorylated/ubiquitinated IκBα. [file 12885_2020_6896_MOESM1_ESM.pdf]

Fig. S1

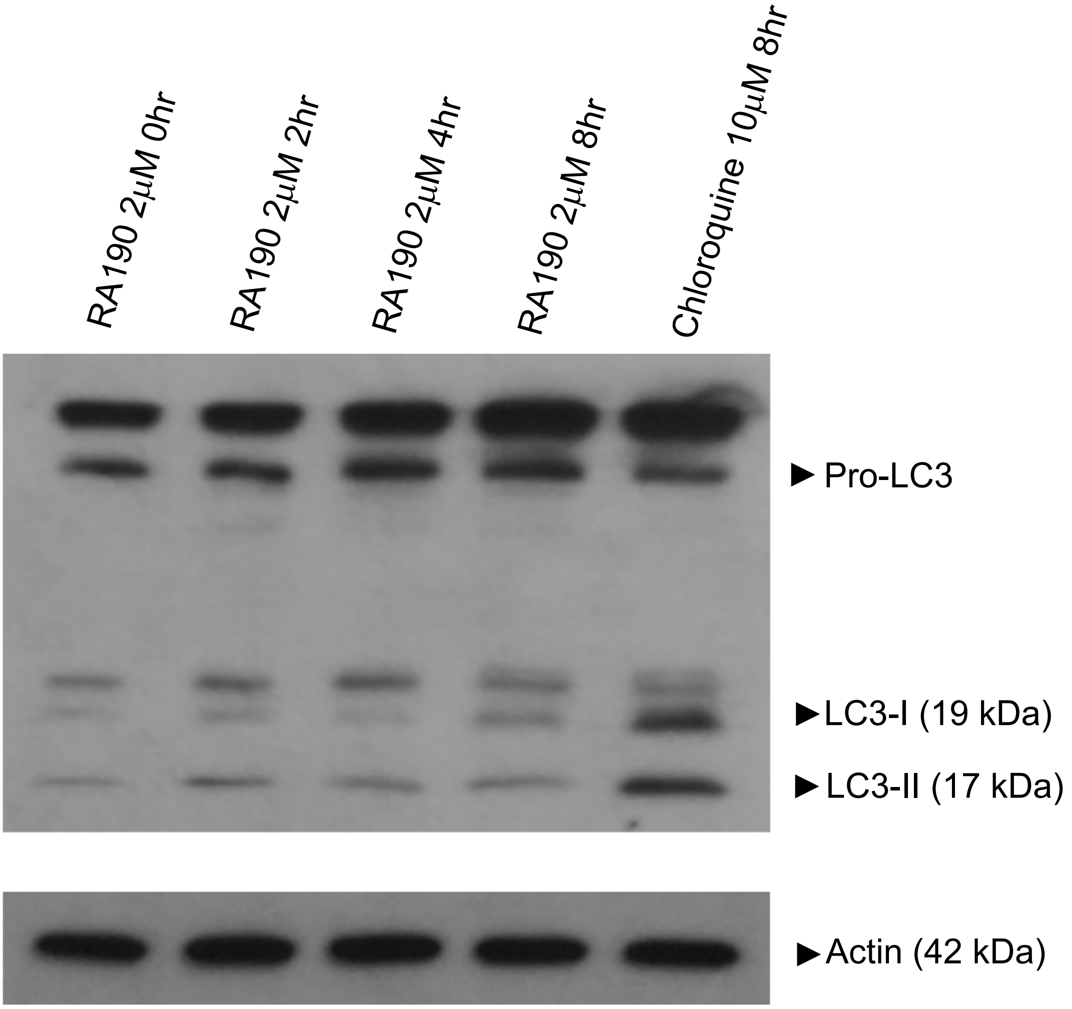

Figure S1. RA190 treatment HepG2 cell lines did not activate the autophagy pathway. HepG2 cells treated with 2 μM RA190 or 10 μM chloroquine (positive control) for 8 hr and then lysates were analyzed by Western blot with antibody to LC3. The lipidated LC3-II was not elevated within 8 hr in the RA190 group (full-length blots/gels are presented in Figure S9) .

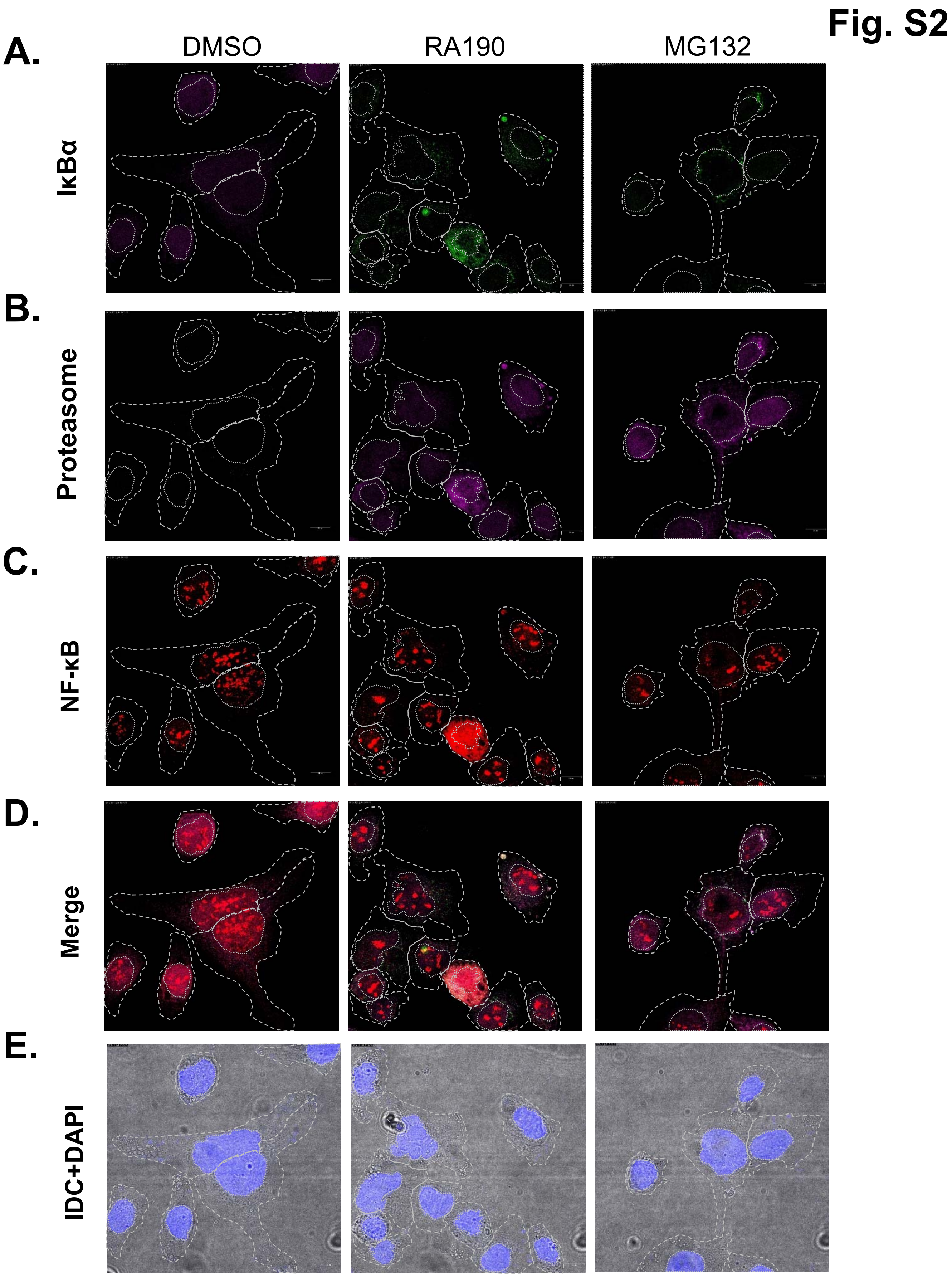

Figure S2. Immunostaining of NF-κB and IκB after treating with RA190. Lower magnification views of Fig.5. The HepG2 cells were treated with DMSO, 2 μM RA190 or 25 μM MG132 for 30 min, and stained for IκBα (A), proteasome (B), or NF-κB (C), and viewed by confocal fluorescence microscopy individually, or (D) merged, or (E) under phase contrast

Fig. S3

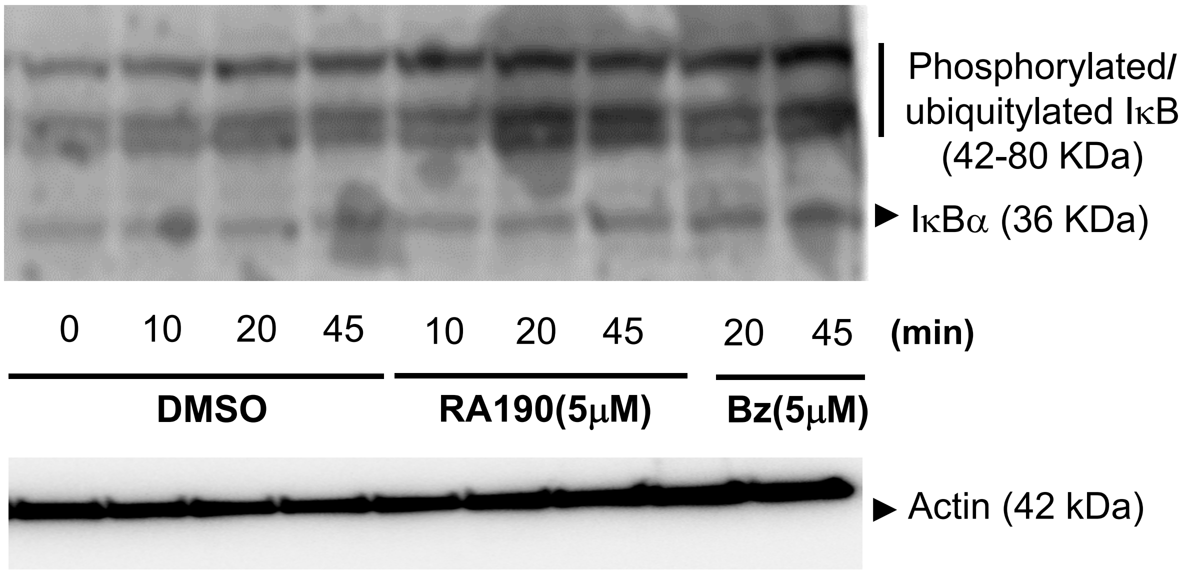

Figure S3. RA190 treatment of HepG2 cells produced significant accumulation of phosphorylated/ubiquitinated IκBα. HepG2 cells were treated by 5 μM RA190, 5 μM bortezomib and DMSO for 0, 10, 20 and 45 min, harvested, lysed and each sample was subjected to Western blot with antibody to IκB and Actin. The phosphorylated/ ubiquitinated IκBα accumulated in RA190 and bortezomib-treated cells (full-length blots/gels are presented in Figure S10).
