# Supplementary material for: Bis-benzylidine Piperidone RA190 treatment of hepatocellular carcinoma via binding RPN13 and inhibiting NF-κB signaling
Source: BMC Cancer. 2020 May 6;20:386. doi: 10.1186/s12885-020-06896-0 (PMC7201939; doi:10.1186/s12885-020-06896-0)
Supplement: Supplementary file 2 — Additional file 2: Figure S4. Original images to Fig. 1C. Figure S5. Original images to Fig. 1D. Figure S6. Original images to Fig. 2A. Figure S7. Original images to Fig. 6A. Figure S8. Original images to Fig. 6C. Figure S9. Original images to Fig. S1. Figure S10. Original images to Fig. S3. [file 12885_2020_6896_MOESM2_ESM.pdf]

Fig. S4

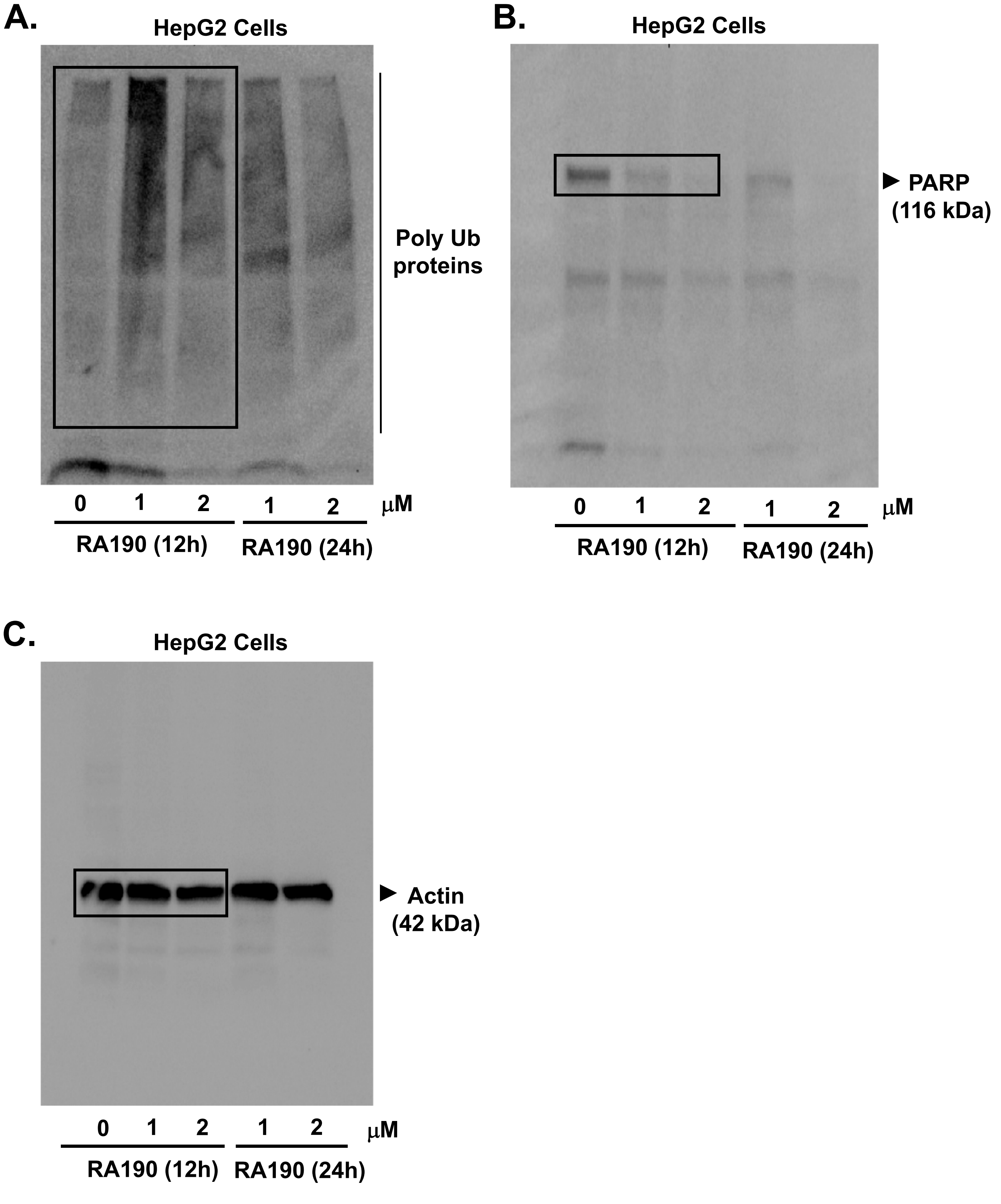

Figure S4. Original images to Figure 1C.
(A) Original image to Figure 1C-Poly Ub proteins. (B) Original image to Figure 1C-PARP. (C) Original image to Figure 1C-Actin.

Fig. S5

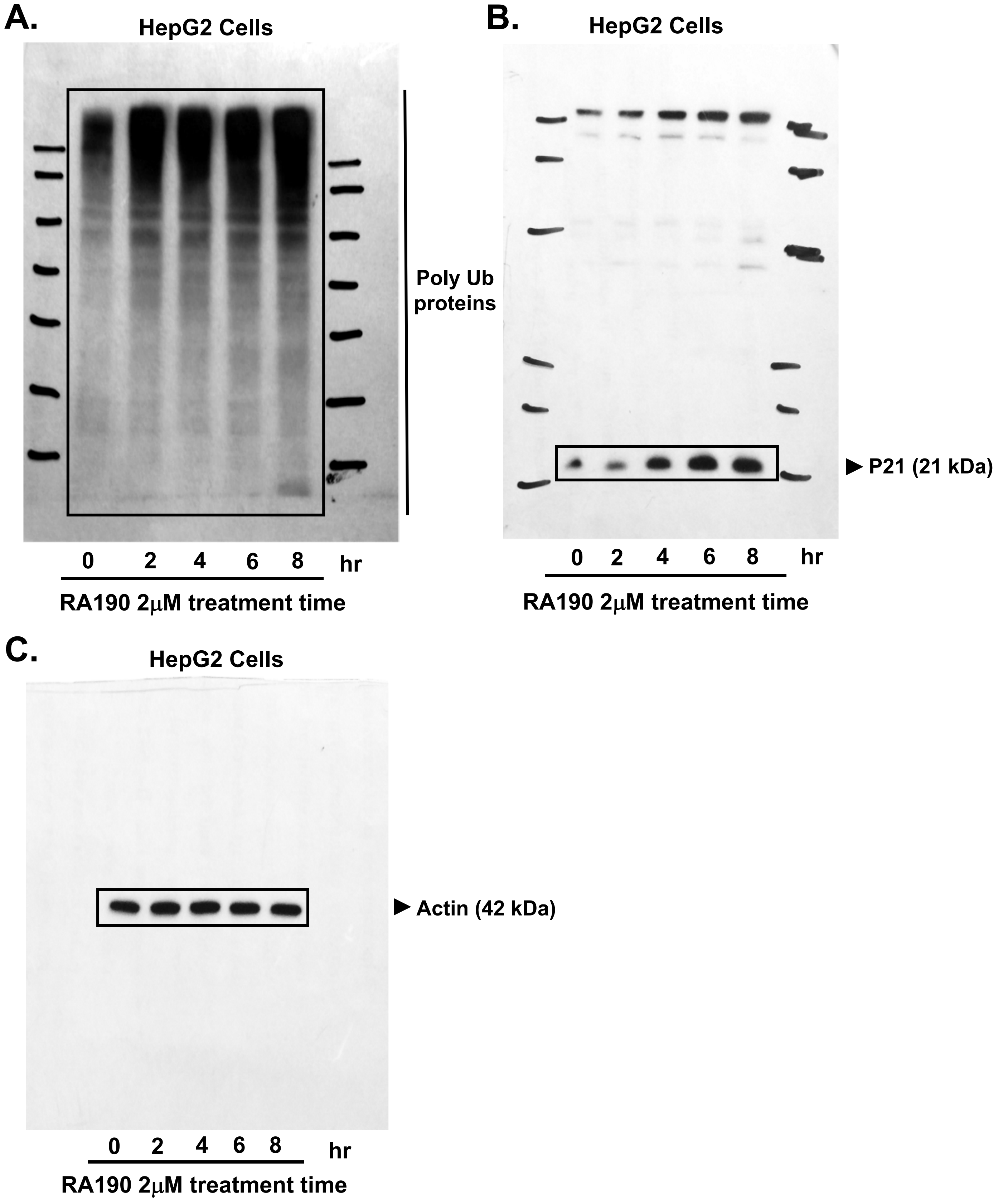

Figure S5. Original images to Figure 1D.
(A) Original image to Figure 1D-Poly Ub proteins. (B) Original image to Figure 1D-P21. (C) Original image to Figure 1D-Actin.

Fig. S6

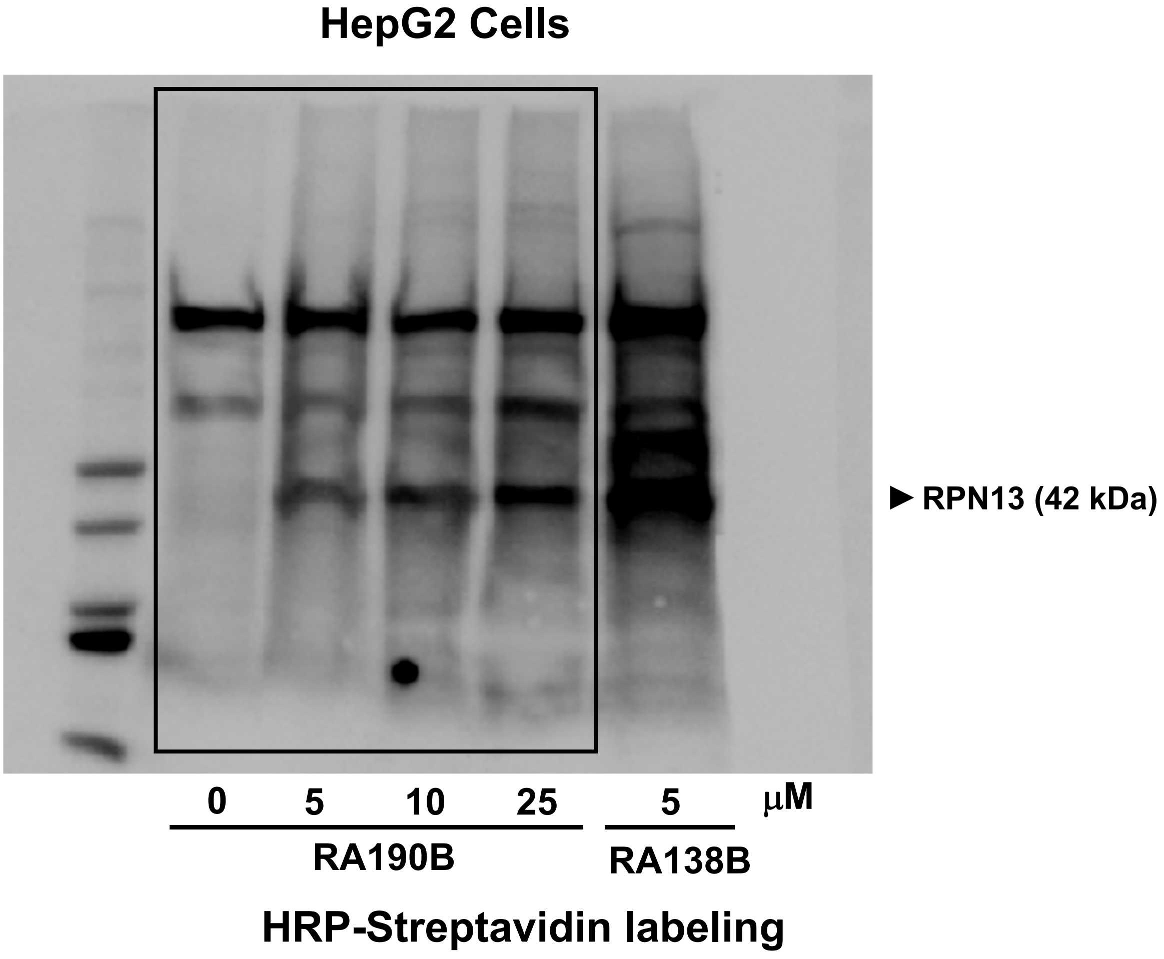

Figure S6. Original images to Figure 2A.
Original image to Figure 2A-RPN13

Fig. S7

A.

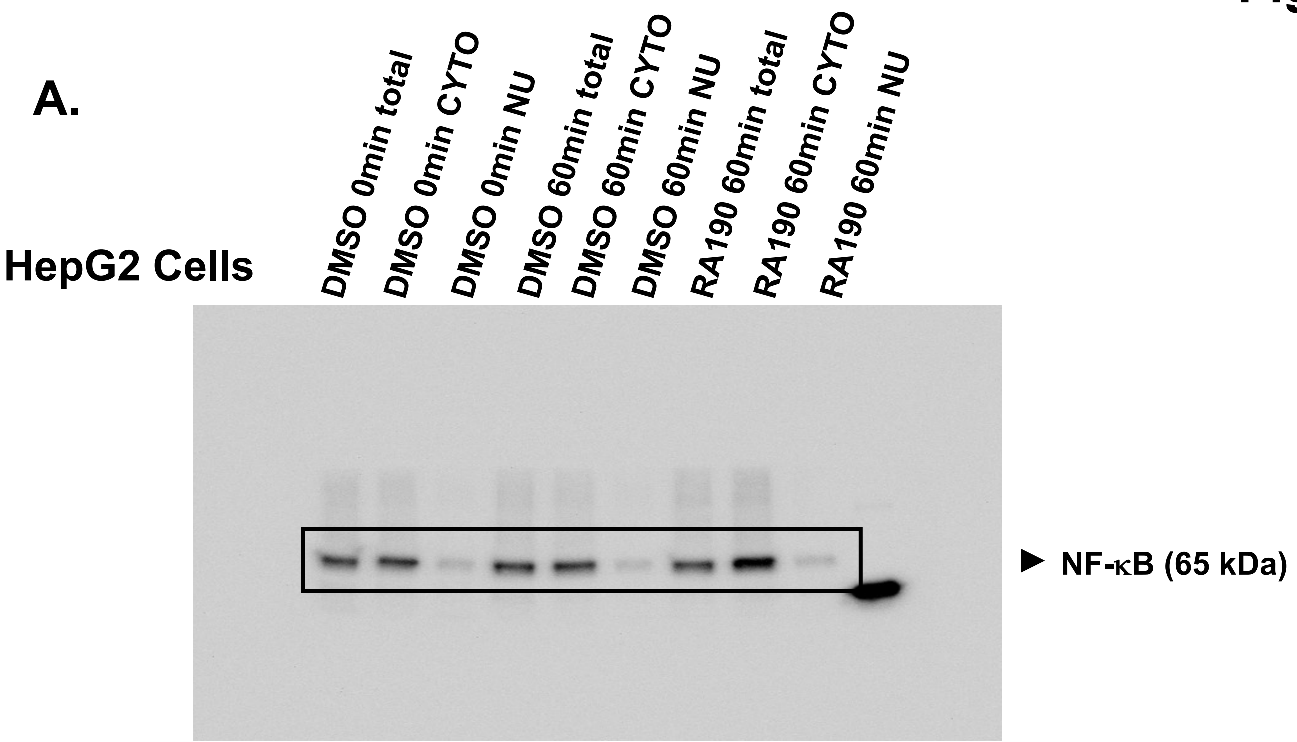

B.

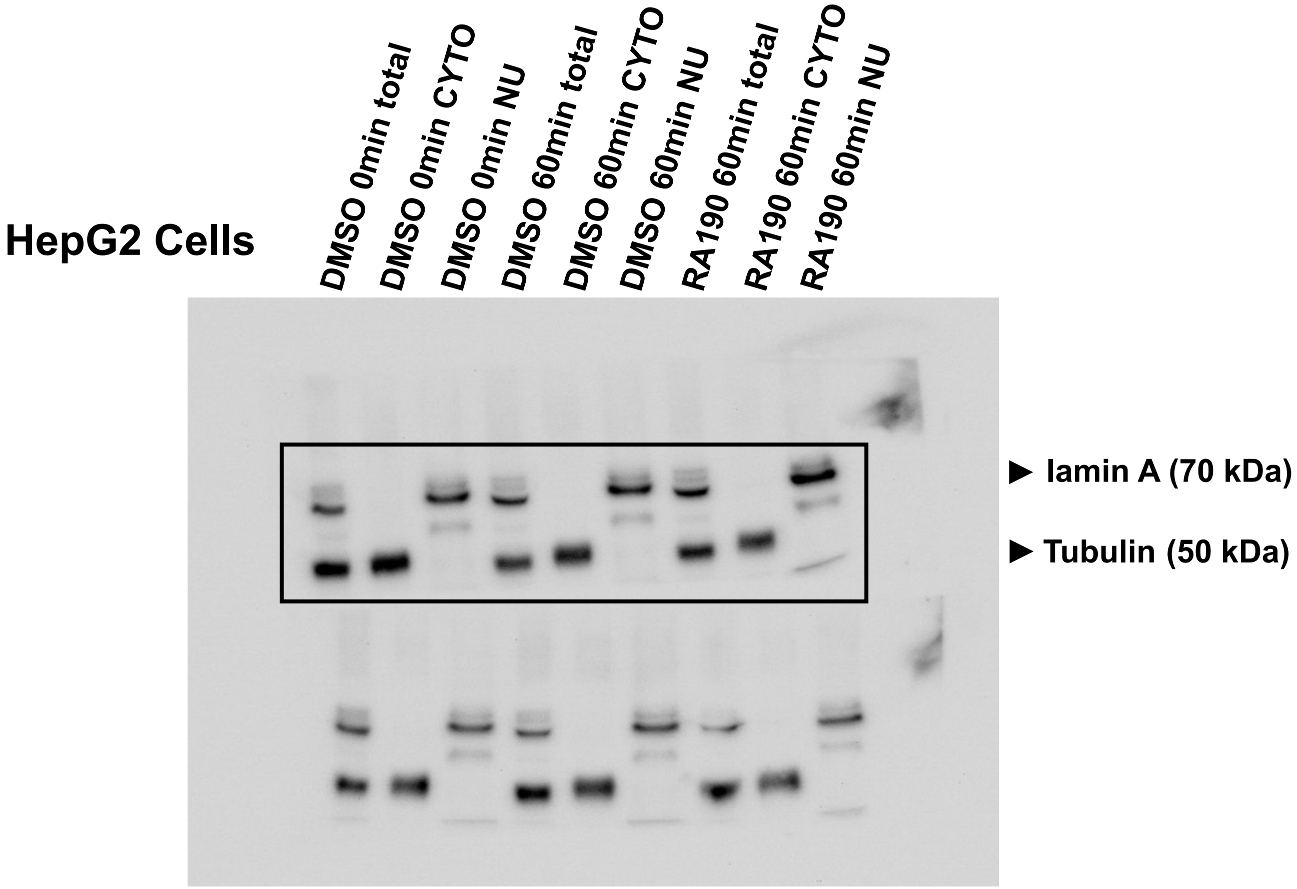

Figure S7. Original images to Figure 6A.
(A) Original image to Figure 6A-NF-κB. (B) Original image to Figure 6A-lamin A and Tubulin.

Fig. S8

A.

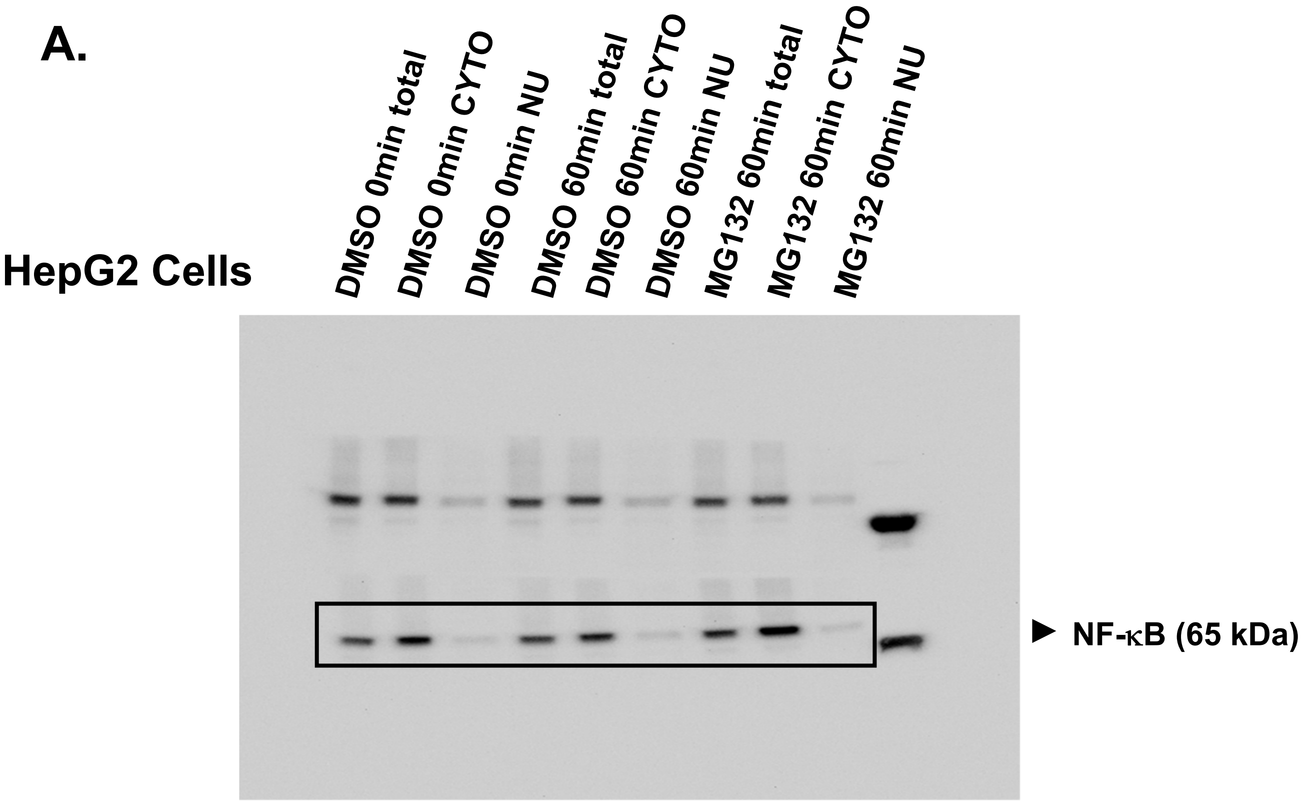

B.

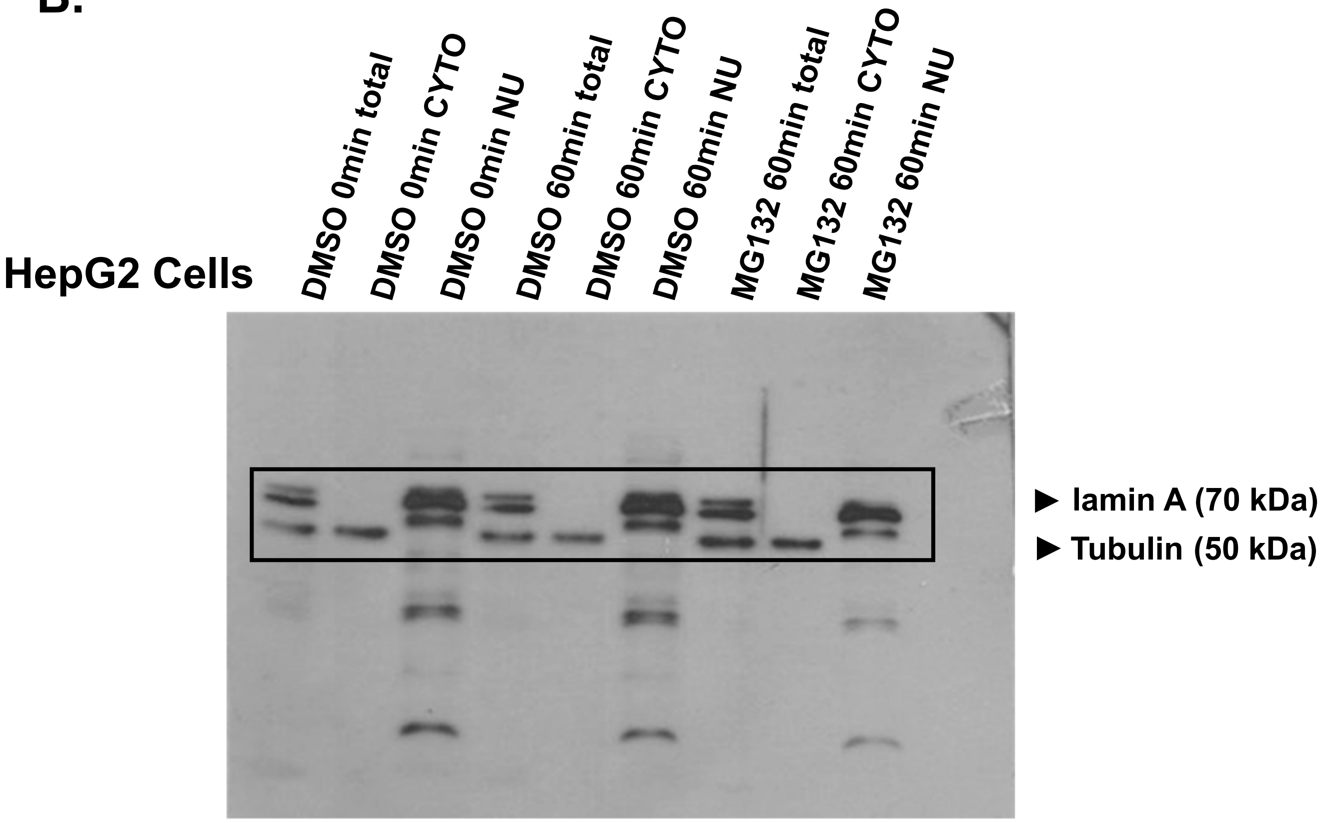

Figure S8. Original images to Figure 6C.
(A) Original image to Figure 6C-NF-κB. (B) Original image to Figure 6C-lamin A and Tubulin.

Fig. S9

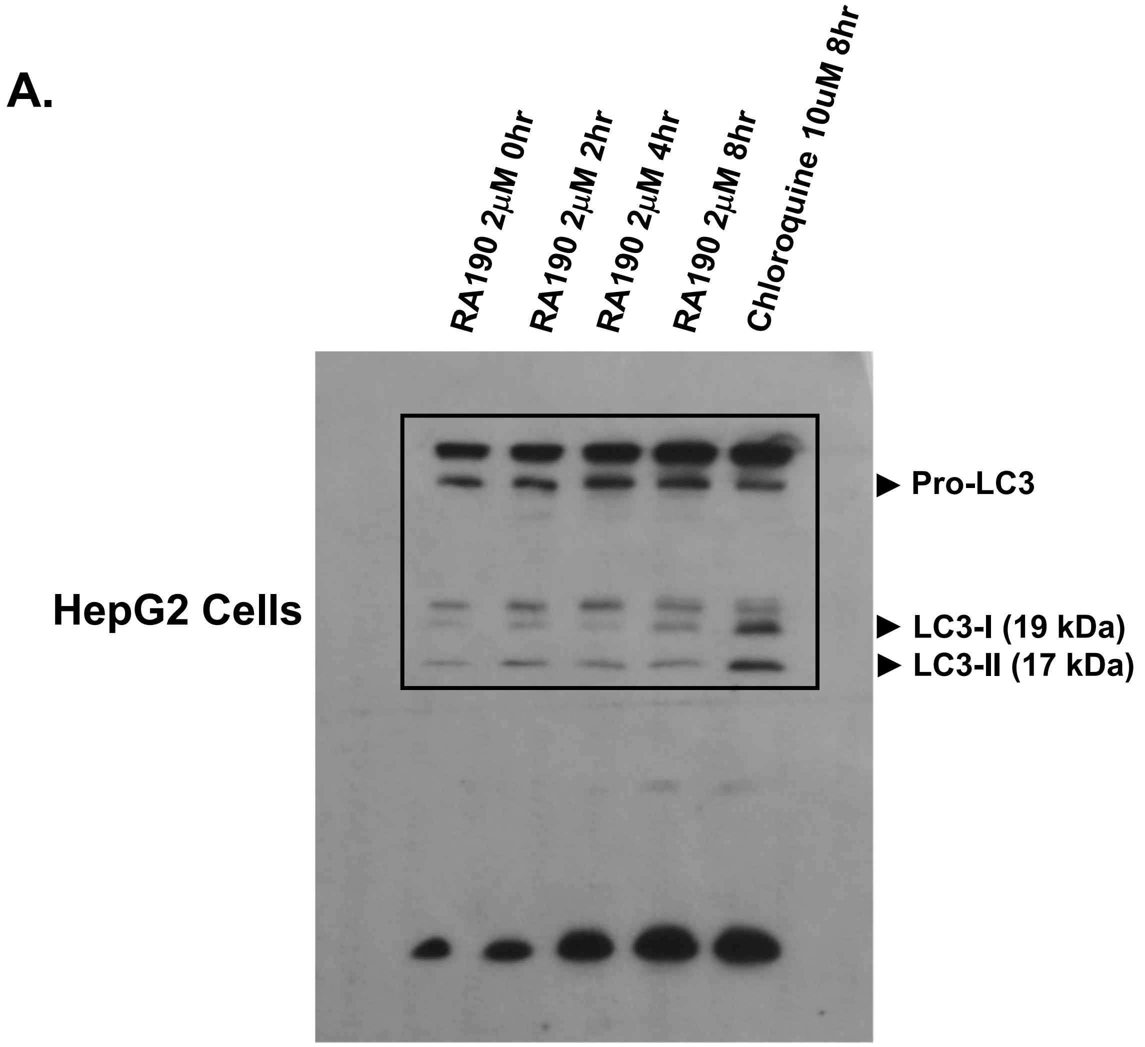

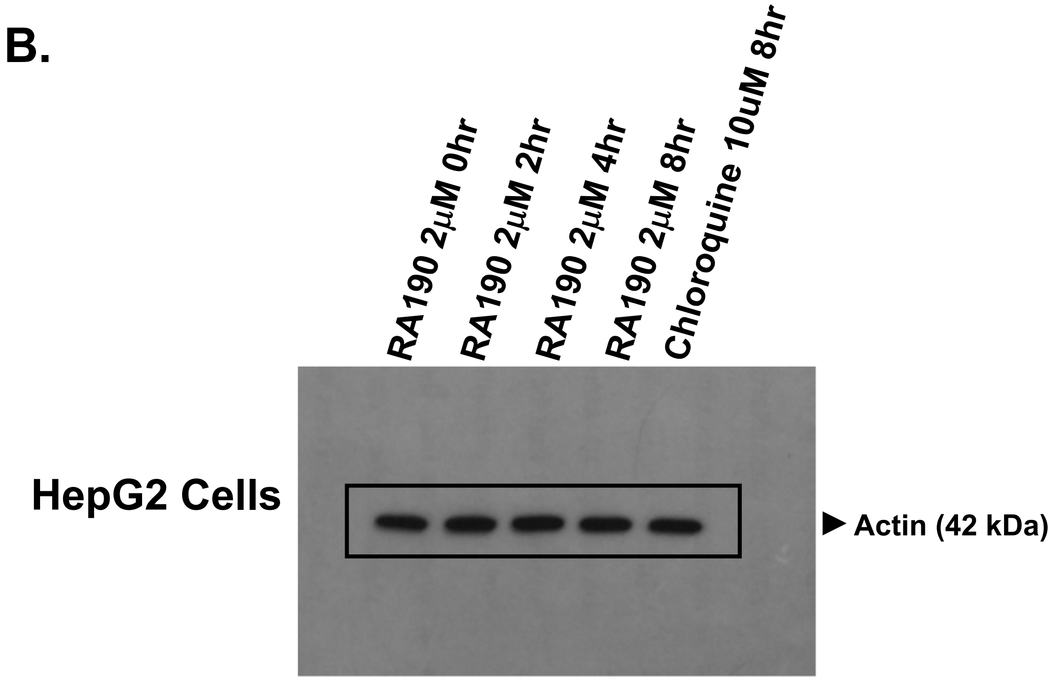

Figure S9. Original images to Figure S1.
(A) Original image to Figure S1-Pro-LC3, LC3-I and LC3-II. (B) Original image to Figure S1-Actin.

Fig. S10

A.

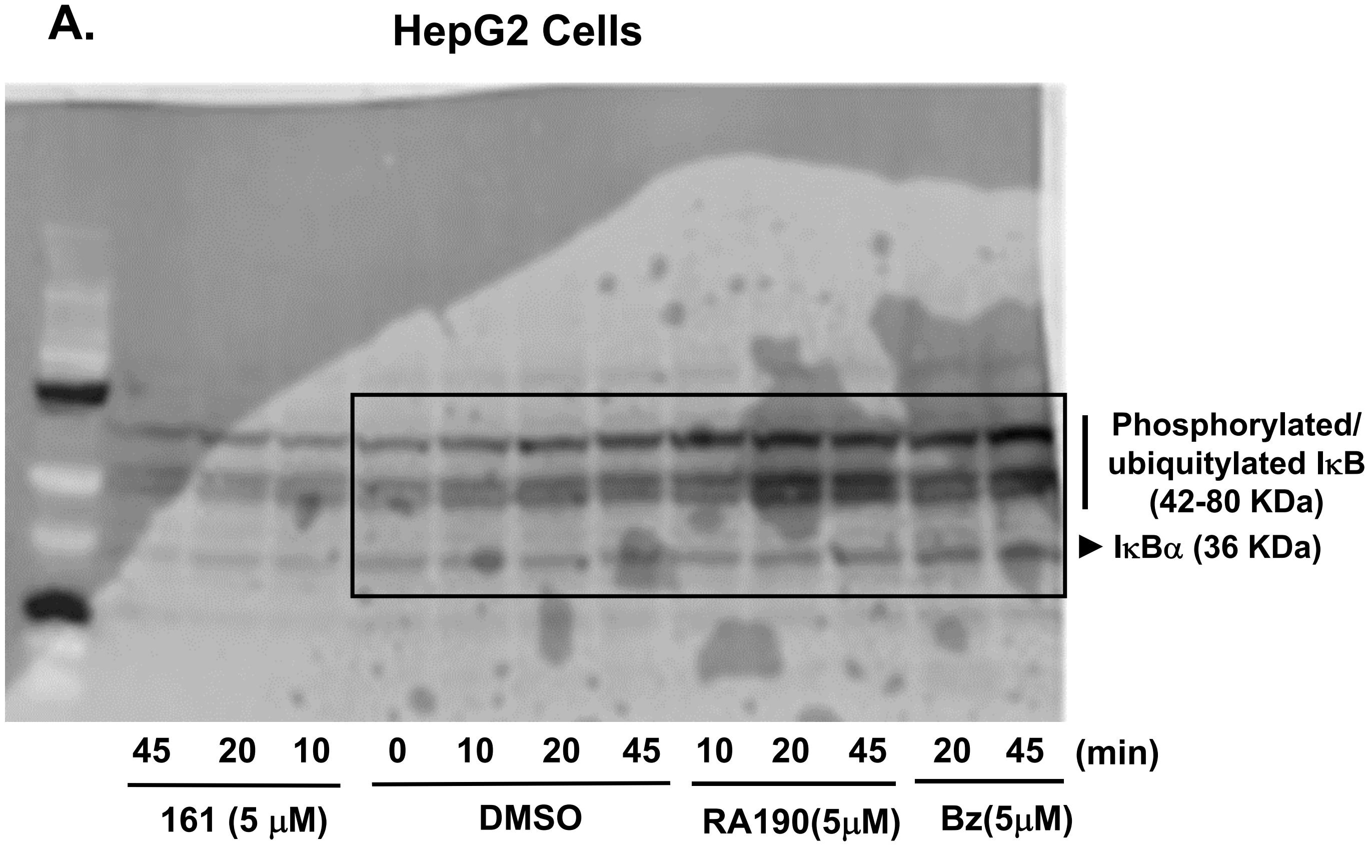

B.

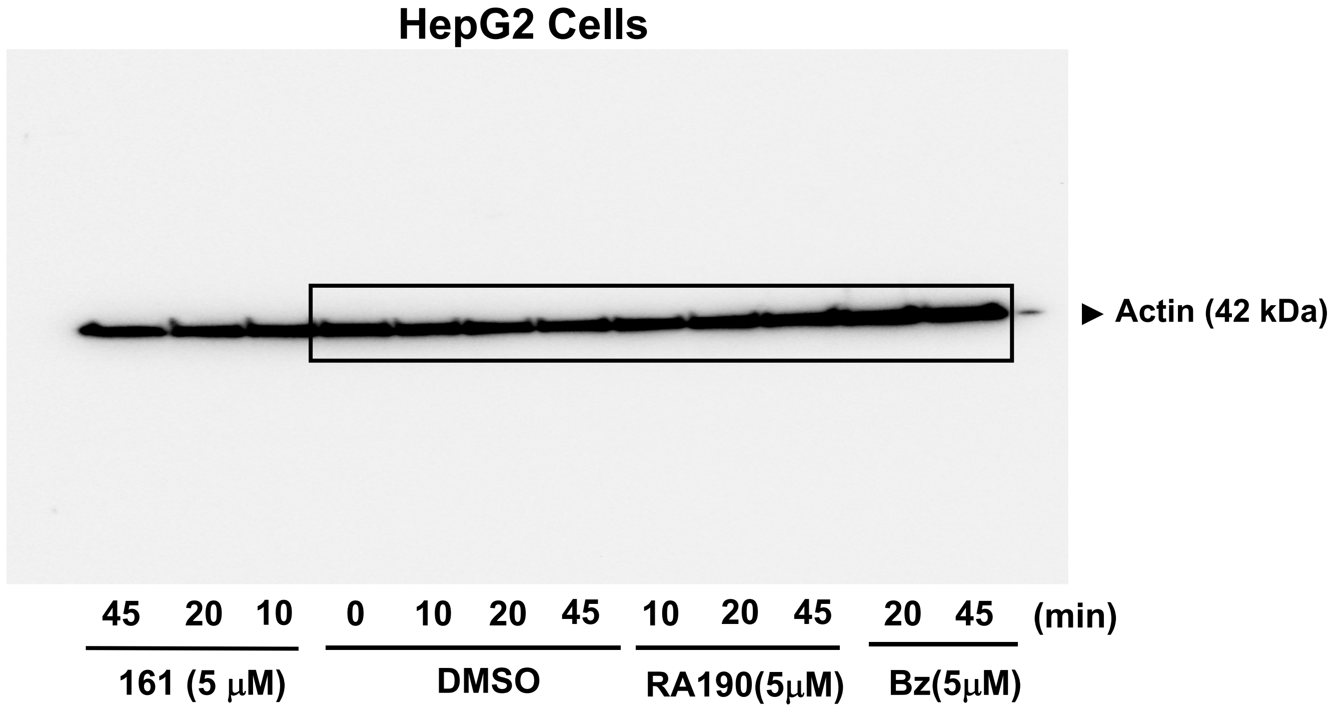

Figure S10. Original images to Figure S3.
(A) Original image to Figure S3-Phosphorylated/ubiquitylated IκB and IκBα. (B) Original image to Figure S3-Actin.
